# Generalization of socially transmitted and instructed avoidance

**DOI:** 10.3389/fnbeh.2015.00159

**Published:** 2015-06-18

**Authors:** Gemma Cameron, Michael W. Schlund, Simon Dymond

**Affiliations:** ^1^Experimental Psychopathology Laboratory, Department of Psychology, Swansea UniversitySwansea, UK; ^2^Department of Behavior Analysis, University of North TexasDenton, TX, USA

**Keywords:** instructed-learning, observational-learning, avoidance, generalization, fear-conditioning, anxiety disorders

## Abstract

Excessive avoidance behavior, in which an instrumental action prevents an upcoming aversive event, is a defining feature of anxiety disorders. Left unchecked, both fear and avoidance of potentially threatening stimuli may generalize to perceptually related stimuli and situations. The behavioral consequences of generalization mean that aversive learning experiences with specific threats may lead to the inference that classes of related stimuli are threatening, potentially dangerous, and need to be avoided, despite differences in physical form. Little is known however about avoidance generalization in humans and the learning pathways by which it may be transmitted. In the present study, we compared two pathways to avoidance—instructions and social observation—on subsequent generalization of avoidance behavior, fear expectancy and physiological arousal. Participants first learned that one cue was a danger cue (conditioned stimulus, CS+) and another was a safety cue (CS−). Groups were then either instructed that a simple avoidance response in the presence of the CS+ cancelled upcoming shock (instructed-learning group) or observed a short movie showing a demonstrator performing the avoidance response to prevent shock (observational-learning group). During generalization testing, danger and safety cues were presented along with generalization stimuli that parametrically varied in perceptual similarity to the CS+. Reinstatement of fear and avoidance was also tested. Findings demonstrate, for the first time, generalization of socially transmitted and instructed avoidance: both groups showed comparable generalization gradients in fear expectancy, avoidance behavior and arousal. Return of fear was evident, suggesting that generalized avoidance remains persistent following extinction testing. The utility of the present paradigm for research on avoidance generalization is discussed.

Chronic or excessive avoidance behavior, in which an overt action postpones or prevents an upcoming aversive event, is a defining feature of anxiety disorders (Craske et al., 2009). In the laboratory, avoidance learning is usually studied within the fear-conditioning paradigm (Dymond and Roche, 2009; Vervliet and Raes, 2013). Fear conditioning involves an initially neutral stimulus (the conditioned stimulus or CS) being paired with an aversive unconditioned stimulus (US), such as electric shock. After only a few CS−US pairings, presentations of the CS alone will elicit a conditioned fear response (CR), measured in humans via physiological arousal, expectancy ratings or action tendencies. Moreover, performing a simple motor response in the presence of the CS that predicts US delivery (CS+) might lead to acquisition of steady rates of avoidance behavior because doing so successfully prevents contact with the US, while rates of avoidance will be low or zero in the presence of the CS that predicts absence of the US (CS−). Several decades of research have been conducted on fear and avoidance learning using variants of this basic paradigm (Boddez et al., 2014; LeDoux, 2014).

A direct instrumental/operant learning history with an avoidance response preventing upcoming US delivery through trial and error may not actually be necessary to learn avoidance. Little is known however about these so-called alternative pathways by which avoidance may be acquired in adults, and to date, much of the basic research has focused on fear learning. Rachman (1977) proposed several vicarious learning pathways to fear other than directly experienced CS−US pairings, such as verbal instructions, in which participants are instructed about the CS−US pairings, and social observation, in which participants observe another individual experience the CS−US pairings. Olsson and Phelps (2004) compared fear learning acquired through direct (CS−US pairings) and indirect experience (verbal instructions and social observation). Participants in the observational-learning group observed a demonstrator’s fearful expression when receiving shocks paired with the angry face CS+, while those in the instructed-learning group were simply informed that the CS+ would be paired with shock. Results showed similar levels of fear learning across all three groups, as measured by skin conductance response (SCR), and similar studies have replicated and extended this basic effect (e.g., Olsson and Phelps, 2007; Raes et al., 2014; Golkar et al., 2015; Mertens et al., 2015). Vicarious learning of fear may help explain how fear is acquired in common childhood fears (e.g., Askew and Field, 2007, 2008; Muris and Field, 2010) and is consistent with the clinical observation that individuals with anxiety do not always report prior direct conditioning episodes like those modeled in fear-conditioning paradigms (Merckelbach et al., 1989; Ollendick and King, 1991).

The evidence to date therefore indicates that both fear and avoidance learning can occur through indirect learning pathways of the kind proposed by Rachman (Field et al., 2001; Askew and Field, 2007; Kelly et al., 2010; Muris and Field, 2010). Avoidance has, however, tended to be measured as a behavioral output of fear, and remains relatively under-investigated in its own right. Indeed, few studies have compared the vicarious pathways through which an avoidance response may be initially acquired. Preliminary evidence for the idea that avoidance may in fact be acquired via alternative pathways was found by Dymond et al. (2012), who tested whether avoidance acquired indirectly via verbal instructions results in similar levels of avoidance behavior and expectancy of shock to avoidance acquired after direct instrumental learning. Following fear conditioning, participants either learned or were instructed to make a response that cancelled upcoming shock. Three groups were then tested with presentations of a directly learned CS+ and CS− (learned group) or instructed CS+ (instructed group). Results showed similar levels of avoidance behavior and expectancy ratings across each of the pathways despite the different routes (i.e., experience vs. instructions) through which they were acquired. These preliminary findings are important because the fear conditioning history with the same danger and safety cues was common across the different pathways; the groups only differed by how the instrumental-avoidance response was acquired before it was subsequently tested under extinction.

No two situations are ever the same, and fear and avoidance acquired in one setting or situation may generalize to perceptually related situations. Generalization of conditioned fear based on formal, perceptual similarity is relatively well studied in humans (Dymond et al., in press) and nonhumans (Kheirbek et al., 2012). Drawing on classic studies of stimulus generalization in nonhumans (Honig and Urcuioli, 1981), systematic tests of fear generalization present an array of stimuli that vary along a specifiable physical continuum (e.g., color or size) from the CS+ (Dunsmoor and Paz, 2015). Generalization of fear and avoidance is adaptive when elicited by stimuli with a high probability of threat. However, the behavioral consequences of fear and avoidance generalization mean that aversive learning experiences with one cue may lead people to infer that classes of related cues are fearful, potentially threatening and need to be avoided, despite differences in physical form. If left unchecked, the focus of fear soon becomes excessive and can lead to debilitating anxiety, impaired social functioning and diminished quality of life. Indeed, the unrestricted generalization or “overgeneralization” of maladaptive fear and avoidance is now widely considered to be a defining feature of anxiety disorders (American Psychiatric Association, 2013). Overgeneralization of conditioned fear has been observed in panic disorder (Lissek et al., 2010), generalized anxiety disorder (Lissek et al., 2014; Tinoco-González et al., in press) and post-traumatic stress disorder (Lissek and Grillon, 2012). Yet, surprisingly little research has been conducted on the generalization of avoidance with healthy participants (Lommen et al., 2010; van Meurs et al., 2014; see also, Geschwind et al., 2015). Lommen et al. (2010) first identified participants who scored high and low for neuroticism and then used white and black colored circles as CS+ and CS−, respectively. During the generalization test, circles with grey values that ranged between black and white were presented as generalization stimuli (GSs) and participants were informed that shocks could be avoided within a latency of 1 or 5 s. Findings showed that participants who scored high on neuroticism only avoided shocks on the 5 s trials compared to the group scoring low on neuroticism.

Recently, van Meurs et al. (2014) devised a “virtual farmer” task to investigate the inter-relationship between Pavlovian fear learning, a passive-emotional process, and the operant/instrumental avoidance it motivates, which may be considered the active-behavioral component of maladaptive coping. The participants’ task was to plant and harvest crops by selecting one of two routes to the field that differed in the likelihood of a successful harvest and the delivery of shock. In the course of the task, circles of differing size (Lissek et al., 2008) appeared onscreen and predicted the delivery of shock during Pavlovian fear and instrumental avoidance generalization trials. During the instrumental avoidance trials, participants had to choose between taking either the short route, which always resulted in a successful harvest but was followed by shock on CS+ trials, or taking the long route, which was never followed by shock but resulted in a reduced likelihood of a successful harvest. Avoidance in the presence of the CS+, by taking the long route, is considered adaptive because it prevents shock, but the extent to which the GSs evoked a maladaptive generalized avoidance tendency was the focus of investigation. van Meurs et al. (2014) found generalization in risk ratings and fear potentiated startle EMG responses obtained on Pavlovian generalization trials and in the proportion of avoidance responses made on instrumental generalization trials. van Meurs et al. (2014) determined patterns of overgeneralized maladaptive avoidance by plotting their measures along a continuum from the CS− via the GSs to the CS+. Similar to studies on the generalization of conditioned fear (Lissek et al., 2005, 2008, 2014), participants’ avoidance behavior resembled a generalization gradient in which conditioned responding reached a maximum in the presence of the CS+, declined as the GSs gradually became more dissimilar, and reached a minimum in the presence of the CS− and a physically unrelated safety cue. Generalization gradients presented in this manner allow for an examination of the strength of generalization by charting the steepness of the gradient: the less steep the gradient, the greater the generalization.

While generalization gradients have been used to directly compare overgeneralization of fear in healthy participants and individuals with clinical disorders (Lissek, 2012), little is known about the overgeneralization or otherwise of avoidance behavior and the mechanisms by which it may be learned and generalized. Studies conducted to date have tended to employ avoidance behavior as a discrete measure of either the motivative properties of fear or as an instantiation of fear itself. For instance, van Meurs et al. (2014) only tested avoidance once in a generalization phase that interspersed Pavlovian fear learning and generalization trials with instrumental-avoidance generalization trials because they were interested in the relationship between passive-emotional Pavlovian and active-behavioral instrumental avoidance. Overlooking the acquisition of avoidance as a signaled operant response (Hurwitz et al., 1972; Higgins and Morris, 1984) may limit our understanding of how maladaptive avoidance coping first comes to be established before it subsequently generalizes and which may then appear to be partially independent of the contribution of Pavlovian processes or not necessitate the simultaneous probing of Pavlovian and instrumental components.

In the present study, we sought to investigate the generalization of signaled operant avoidance following a direct Pavlovian fear learning history in which one cue was established as a danger cue (CS+) and another as a safety cue (CS−). We then compared different routes or pathways by which the avoidance response is learned on subsequent generalization. Our aim was to contrast instructed-learning and observational-learning pathways of generalized avoidance. Following preacquisition and fear conditioning phases, groups were either instructed that a simple instrumental response in the presence of the CS+ cancels upcoming shock or observed a short movie showing a demonstrator in the same experimental context performing the avoidance response to prevent shock. In the generalization test phase, learned danger and safety cues were presented along with generalization stimuli (GS_1_, GS_2_, GS_3_) in the absence of the US (extinction), and avoidance behavior, US expectancy and SCR measured.

In addition, we then sought to test whether, after the end of the extinction test block, three unsignaled US presentations would prompt reinstatement of generalized avoidance if the generalization test was repeated. Reinstatement tests like this model the real world return of fear that often interferes with the long-term effectiveness of exposure-based therapy (Haaker et al., 2014). Interestingly, reinstatement studies with humans have shown a post-extinction increase in outcome measures to the CS+ and also the generalization of reinstatement effects to the CS− (Kull et al., 2012). To our knowledge, reinstatement of generalized avoidance has not been tested before in humans. A secondary aim of the present study was therefore to investigate the effects of reinstatement on generalized avoidance both in terms of the physically similar stimuli resembling CS+ and the safety cue, CS−.

We hypothesized higher trial-by-trial US expectancy ratings, avoidance behavior and SCRs to CS+ than CS−, and generalization of these outcome measures to stimuli that are more physically similar to the CS+ than CS−. We also hypothesized that there would be no differences in outcome measures during extinction testing between individuals who have acquired avoidance via either instructed-learning or observational-learning. Moreover, we hypothesized that reinstatement testing following extinction would result in less steep gradients overall to GSs arranged along the physical continuum between CS− and CS+ in both groups. Given that the present study predicted a degree of equivalence between the instructed-learning and observational-learning pathways, conventional null hypothesis testing is somewhat limited. For this reason, and because a non-significant *p*-value does not provide support for the null hypothesis, we used an additional, Bayesian analysis to establish the statistical likelihood of the null hypotheses being valid over the alternative hypothesis. The Bayesian framework has several theoretical advantages over classical frequentist statistics (Dienes, 2014), which allows us to quantify the probability of the null hypothesis being true (Wagenmakers, 2007).

## Materials and Methods

### Participants

Fifty-four healthy participants, 15 men and 39 women (*M*_age_ = 20.13 years, *SD* = 3.30) without a self-reported history of anxiety or depression, were randomly assigned to one of two groups: Instructed-learning or Observational-learning. All participants provided informed consent and were compensated with either course credits or the opportunity to win a £15 voucher. The Department of Psychology Ethics Committee at Swansea University approved the study and all procedures were conducted in accordance with the Declaration of Helsinki for the protection of human participants.

### Apparatus and Stimuli

**Figure 1:**
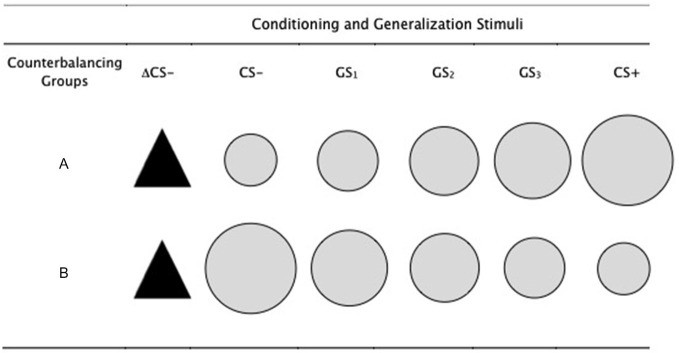
**Conditioning and generalization stimuli**. The conditioned stimuli (CS+ and CS−) were counterbalanced across participants, such that for half of the participants (group A) the CS+ was the largest of the circles and CS− was the smallest. This was reversed for the other half (group B). The generalization stimuli were circles of intermediate sizes between CS+ and CS−, gradually increasing in size for group B and gradually decreasing in size for group A. The ΔCS− was the same for all participants. The smallest circle had a diameter of 5 cm, increasing progressively in size by 15% for each stimulus, such that the second smallest circle was 15% larger than the first and 15% smaller than the next (i.e., 5 cm, 5.8 cm, 6.6 cm, 7.6 cm, 8.7 cm). The ΔCS− had a width and height of 6.6 cm, comparable to that of the GS_2_, which also remained the same across groups.

Five gray colored circles of increasing size were used as the conditioned and generalization stimuli, with the largest and smallest circles serving as the CS+ and CS−, in a counterbalanced order of conditions (see Figure 1). The remaining three circles served as the generalization stimuli (GS_1_, GS_2_, GS_3_). The smallest circle had a diameter of 5 cm, increasing progressively in size by 15% for each stimulus, such that the second smallest circle was 15% larger than the first and 15% smaller than the next (i.e., 5 cm, 5.8 cm, 6.6 cm, 7.6 cm, 8.7 cm). A black isosceles triangle served as a perceptually dissimilar novel safety cue (ΔCS− had a width and height of 6.6 cm, comparable to that of the GS_2_, which also remained the same across groups. Stimuli were presented on a 17″ computer monitor with a 60 Hz refresh rate, and the stimulus sequence, presentation and timings were controlled using *Open Sesame* (Mathôt et al., 2012).

Electric shock (250 ms duration) was delivered via a bar electrode fitted to the participant’s dominant forearm and controlled by an isolated stimulator (STM200–1, BIOPAC Systems, Santa Barbara, CA, USA). At the outset, all participants underwent a shock calibration procedure in which they were given an example shock and instructed to either select or retain a shock level that was “uncomfortable, but not painful”. The shock level selected by each participant was used throughout the experiment. SCRs were acquired from the distal phalanx of the second and third digits on the non-dominant hand and recorded using the BIOPAC MP-150 SCR module (BIOPAC Systems, Santa Barbara, CA, USA). Isotonic recording gel was applied to the Ag-AgCl 4 mm electrodes prior to their application.

### Procedure

Following informed consent, participants were fitted with the SCR and shock electrodes and undertook shock calibration. Participants were then given general procedural instructions explaining that on each trial one of two colored circles would appear, that some may be followed by shock, and that when the US expectancy rating questions appeared on screen they should use the mouse to rate the likelihood of shock (where *0 = certainly no shock, 5 = uncertain and 10 = certainly shock*).

The procedure consisted of six phases: *preacquisition, fear conditioning, avoidance learning, generalization test, US reinstatement*, and* reinstatement test* (see Table 1). Both groups experienced all phases, but contingencies differed between-groups in the avoidance learning phase only.

**Table 1:** **Trial types and number of stimulus presentations during preacquisition, fear conditioning, avoidance learning, generalization test, and reinstatement test phases**.

	Conditioned and generalized stimuli						
						CS+	
Phase	ΔCS−	CS−	GS_1_	GS_2_	GS_3_	US?	No US?
Preacquisition		1					1
Fear conditioning		6				6
Avoidance learning		8				4*	4
Generalization test	2	4	4	4	4		4
Reinstatement test	2	4	4	4	4		4

Note: Three unsignaled US presentations occurred between the Generalization Test and Reinstatement Test phases. “US?” and “NoUS?” ask whether shock was or was not presented. *Indicates that the US occurred on unavoidable trials (for the Instructed-learning group only).

#### Preacquisition

The CS+ and CS− were each presented once in the center of the screen for 2 s followed by the rating scale, which remained on screen until a response occurred or for a maximum of 4 s, whichever happened first. The CS duration was therefore 6 s and the intertrial interval (ITI), which varied between 6 s and 8 s, was indicated by a black fixation cross. No shocks were presented in this phase.

#### Fear Conditioning

In this phase, which continued uninterrupted following the previous ITI, CS+ and CS− were each presented six times in a randomized order (CS duration was 6 s). The termination of every CS+ trial (either by a rating response or by reaching its maximum duration) was always followed by shock. No shocks ever followed the CS−.

#### Avoidance Learning

**Figure 2:**
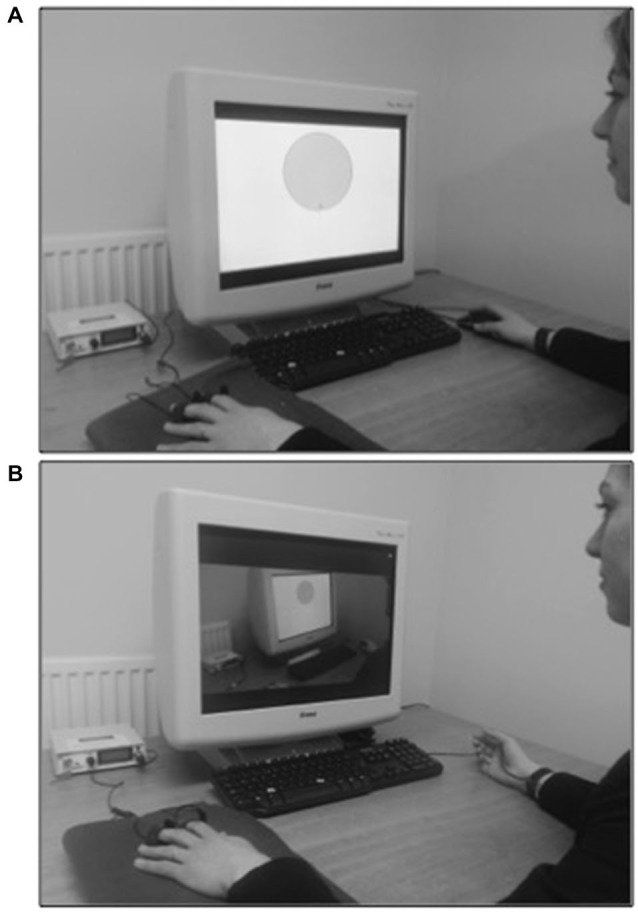
**Instructed-learning and observational-learning of avoidance**. After fear conditioning, an individual was instructed how and when to perform the avoidance response (mouse click) in the presence of CS+ **(A)** or observed a learning model performing the avoidance response **(B)**.

During this phase, the Instructed-learning group was told that their task was to learn to make a response to prevent shock (Figure 2). They were told that on some trials a black border would appear around the edge of the screen and would signal the availability of the avoidance response, which consisted of pressing the right mouse button once with the cursor hovering over the CS+. Participants were presented with the CS+ and CS− a further eight times; on half of the trials, the avoidance cue was presented, which signaled the availability of the mouse button response (CS duration was 6 s). On the avoidable trials, the stimulus (CS+ or CS−) was presented for 2 s and followed by the avoidance cue for 2 s: during this time the stimuli remained on screen and participants could use the mouse to click on the image to prevent pending shock. Participants made ratings on the US expectancy scale before making or not any avoidance response. On unavoidable trials, the stimulus (CS+ or CS−) was presented for 2 s and, 2 s later, shock always followed CS+ trials only. Shocks never followed any CS− trials. Participants were informed they should only make the avoidance response if they believed that shock would follow the image on the screen and that once the rating scale appeared, the avoidance response, when available, could no longer be performed and that they should instead make a rating on the scale. The mouse cursor was hidden until available to use, either at the onset of the avoidance cue or rating scale.

The Observational-learning group did not take part in any learning trials in this phase. Instead, they viewed a short (4 min) film of a male demonstrator taking part in the avoidance-learning phase of the same experiment (Figure 2). They were told that they would observe a person taking part an experiment similar to the one that they themselves would be taking part in after the video had ended. They were also told that the person in the film would learn to cancel an upcoming shock using the mouse and that they should pay close attention to the screen because they too would have to learn to cancel upcoming shocks. These participants observed a total of 16 trials (i.e., CS+ and CS− each presented eight times) in which the CS+ was always avoided when the border appeared and the CS− never avoided (the demonstrator, but not the participant, received a total of four shocks on CS+ unavoidable trials). The demonstrator made ratings on every trial (which were always high for CS+ unavoidable trials and low for all CS− and CS+ avoidable trials). The Observational-learning group made no ratings during this phase.

#### Generalization Test

This phase continued uninterrupted and without further instructions. The CS+, CS−, GS_1_, GS_2_, and GS_3_ were each presented four times (two avoidable and two unavoidable trials of each cue) along with two presentations of the ΔCS− for a total of 22 trials. The cue signaling an avoidable trial appeared on all CS+, CS− and GS trials, but never on ΔCS− trials. As this was a test phase, shock was withheld on all trials.

#### US Reinstatement

Following a short interval (1250 ms), the US was presented three times without warning and in the absence of any onscreen stimuli. Each US presentation was separated by a delay of 1 s.

#### Reinstatement Test

Finally, a short interval (1 s) commenced before the scheduled ITI and trials were re-presented from the generalization test phase.

### Data Analysis

Skin conductance data were continuously recorded at a rate of 1000 samples per second and off-line analysis of the analog SCR waveforms conducted with AcqKnowledge (BIOPAC Systems Inc., Goleta, CA, USA). SCRs were measured for each trial as the peak-to-peak amplitude difference in SCR to the first response (in microsiemens, μS) in the 0.5–6 s latency window following stimulus onset. The minimal response criterion was 0.02 μS. To normalize the SCR data, scores were square-root transformed. Statistical analysis of SCR data involved repeated- measures ANOVA.

Online US expectancy ratings appeared on every trial for the Instructed-learning group and on all trials excluding the avoidance learning phase for the Observational-learning group. Where participants responded within the time allowed, ratings for each stimulus were analyzed within test phases and separately for avoidable and unavoidable trials. The analysis of US expectancy ratings and SCRs focused on avoidable trials during avoidance learning and test phases. All ΔCS− trials were unavoidable during these phases and were not included in the final analysis. For all phases, excluding the avoidance learning phase, a two-way repeated measures ANOVA was used to compare within and between subject differences for the dependent measures. For the generalization and reinstatement test phases only, a polynominal trend analysis was conducted to determine the linear and quadratic terms used to describe the shape of the generalization gradients obtained (only significant trends are reported). A paired samples *t*-test was used to analyze data from the Instructed-learning group during avoidance learning. Avoidance behavior was measured as a percentage of trials avoided for all avoidable CS+, CS− and GS stimuli. For all analyses, the alpha level was set at 0.05, where necessary, *p*-values reflect the Greenhouse-Geisser correction for sphericity, and Bonferroni correction was used to control for multiple comparisons.

To further investigate the predicted absence of between-group differences, we performed repeated-measures Bayesian ANOVA with JASP (Love et al., 2015) and used default priors to estimate the Bayes Factor (BF; Rouder et al., 2012). The BF indicates the likelihood of the data fitting under the null hypothesis with the likelihood of fitting under the alternative hypothesis. In our analysis, we compared the null hypothesis against the alternative (BF_01_), where the greater the BF value, the greater the likelihood of the data fitting the null hypothesis (e.g., a BF greater than 3 indicates substantial evidence for the null hypothesis, 1 indicates no evidence for either theory, and less than 1 indicates increasing evidence for the alternative hypothesis; Wetzels and Wagenmakers, 2012).

## Results

A total of four participants were removed from all analyses (three from the Instructed-learning group and one from the Observational-learning group) due to a programming error, while a further one participant’s data from the Instructed-learning group was excluded from analysis of the avoidance learning phase only. The final sample sizes were: Instructed-learning (*n* = 25) and Observational-learning (*n* = 25). Of these, SCR data from three participants (one Instructed-learning and two Observational-learning) were removed from the analysis as they were deemed non-responders; due to a programming error, data were missing from a further two participants from each of the groups, respectively, and two further participants from the Observational-learning group were removed from analysis of the reinstatement test phase only because they removed the electrodes.

### Preacquisition

#### US Expectancy Ratings

As expected, ratings of the likelihood of shock did not differ across stimuli during preacquisition, *F*_(1,34)_ = 1.969, *p* = 0.170, ${\text{η}}_{\text{p}}^{\text{2}}$ = 0.055, BF_01_ = 1.945, there was no interaction with group, *F*_(1,34)_ = 0.362, *p* = 0.552, ${\text{η}}_{\text{p}}^{\text{2}}$ = 0.011, BF_01_ = 3.811, and no differences between groups, *F*_(1,34)_ = 0.049, *p* = 0.826, ${\text{η}}_{\text{p}}^{\text{2}}$ = 0.001, BF_01_ = 1.985.

#### SCR

Analysis of SCR revealed a similar pattern, with no differences between stimulus type, *F*_(1,46)_ = 0.468, *p* = 0.497, ${\text{η}}_{\text{p}}^{\text{2}}$ = 0.010, BF_01_ = 3.687, no interaction, *F*_(1,46)_ = 0.569, *p* = 0.454, ${\text{η}}_{\text{p}}^{\text{2}}$ = 0.012, BF_01_ = 13.425, and no differences between groups, *F*_(1,46)_ = 0.049, *p* = 0.827, ${\text{η}}_{\text{p}}^{\text{2}}$ = 0.001, BF_01_ = 3.783.

Table 2 shows the means (and standard deviations) for US expectancy ratings and SCR for CS+ and CS− during Preacquisition, Fear Conditioning, and Avoidance Learning phases (avoidable trials only) for both groups.

**Table 2:** **Means (and standard deviations) for US expectancy ratings and SCR for CS+ and CS- during preacquisition, fear conditioning, and avoidance learning phases (avoidable trials only) for the instructed-learning and observational-learning groups**.

Stimulus	Group	Preacquisition		Fear conditioning		Avoidance learning	
		Ratings	SCR	Ratings	SCR	Ratings	SCR
CS−	Instructed	3.37 (2.36)	1.67 (1.41)	3.29 (2.15)	1.67 (1.40)	1.53 (1.82)	1.25 (1.10)
	Observed	3.35 (2.14)	2.33 (1.71)	3.67 (1.92)	2.33 (1.71)	–	–
CS+	Instructed	4.19 (2.38)	2.08 (1.51)	6.92 (1.72)	2.08 (1.51)	7.58 (2.36)	1.98 (1.25)
	Observed	3.55 (2.15)	2.97 (1.68)	6.80 (1.30)	2.97 (1.68)	–	–

The expectancy ratings and SCR findings were predicted given the absence of shock during preacquisition, and showed that the groups had a similar, low expectancy of shock and undifferentiated SCR profile at the outset.

### Fear Conditioning

#### US Expectancy Ratings

**Figure 3:**
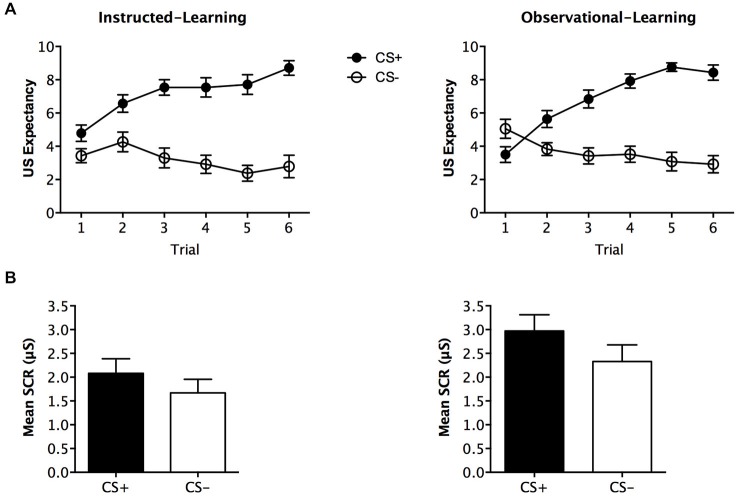
**Fear conditioning results**. Trial by trial unconditioned stimulus (US) expectancy **(A)** and mean skin conductance response (SCR) (μS) (square-root transformed) **(B)** results for CS+ and CS− presentations during fear conditioning for the instructed-learning and observational-learning groups. Error bars indicate SEM.

During fear conditioning, expectancy ratings differed across stimuli, *F*_(1,48)_ = 65.342, *p* < 0.001, ${\text{η}}_{\text{p}}^{\text{2}}$ = 0.577, BF_01_ = 7.007, but no interaction with group was found, *F*_(1,48)_ = 0.374, *p* = 0.544, ${\text{η}}_{\text{p}}^{\text{2}}$ = 0.008, BF_01_ = 2.830. The instructed-learning and observational-learning groups did not differ in their expectancy of shock, *F*_(1,48)_ = 0.200, *p* = 0.657, ${\text{η}}_{\text{p}}^{\text{2}}$ = 0.004, BF_01_ = 4.306. Analysis of trial-by-trial ratings for this phase with trial order as within subjects factor and group as between subjects factor, revealed significantly higher expectancy across trials for CS+ *F*_(5,420)_ = 26.519, *p* < 0.001, ${\text{η}}_{\text{p}}^{\text{2}}$ = 0.356, BF_01_ = 2.388e-19, and which did not differ between the groups *F*_(1,48)_ = 1.235, *p* = 0.272, ${\text{η}}_{\text{p}}^{\text{2}}$ = 0.025, BF_01_ = 3.535. As predicted, this indicates that both groups demonstrated an increase in US expectancy across trials (see Figure 3A).

#### SCR

Analysis of SCR revealed no significant main effect of stimulus, *F*_(1,40)_ = 3.313, *p* = 0.076, ${\text{η}}_{\text{p}}^{\text{2}}$ = 0.076, BF_01_ = 0.973, and no interaction with group, *F*_(1,40)_ = 0.162, *p* = 0.690, ${\text{η}}_{\text{p}}^{\text{2}}$ = 0.004, BF_01_ = 0.749. The groups had a near significant difference in overall SCR, *F*_(1,40)_ = 3.878, *p* = 0.056, ${\text{η}}_{\text{p}}^{\text{2}}$ = 0.088, BF_01_ = 0.781, but were similar in SCRs elicited to CS− (*p* = 0.178) and CS+ (*p* = 0.077; see Figure 3B).

### Avoidance Learning

#### US Expectancy Ratings

The instructed-learning group’s ratings during avoidable, *t*_(23)_ = 10.429, *p* < 0.001, and unavoidable trials, *t*_(23)_ = 10.854, *p* < 0.001, differed. This indicated high expectancy of shock following CS+ than CS−, irrespective of the availability of the avoidance response.

#### Avoidance Behavior

The instructed learning group performed the avoidance response on 73.9% of CS+ trials (*SD*: 37.9) and 25% of CS− trials (*SD*: 38.3). The proportion of avoidance behavior evoked by the cues was significantly different, *t*_(23)_ = 4.579, *p* < 0.001, indicating a higher proportion of avoidance responses made to CS+ compared to CS− during avoidable trials.

#### SCR

The SCR elicited by CS+ and CS− during avoidable, *t*_(20)_ = 2.482, *p* < 0.05, and unavoidable trials, *t*_(21)_ = 2.327, *p* < 0.05, differed, which indicated an increased physiological response to the danger cue (CS+) than the safety cue (CS−) during avoidable and unavoidable trials. Interestingly, the availability of avoidance did not modulate SCRs to the CS+.

### Generalization Test

#### US Expectancy Ratings

**Figure 4:**
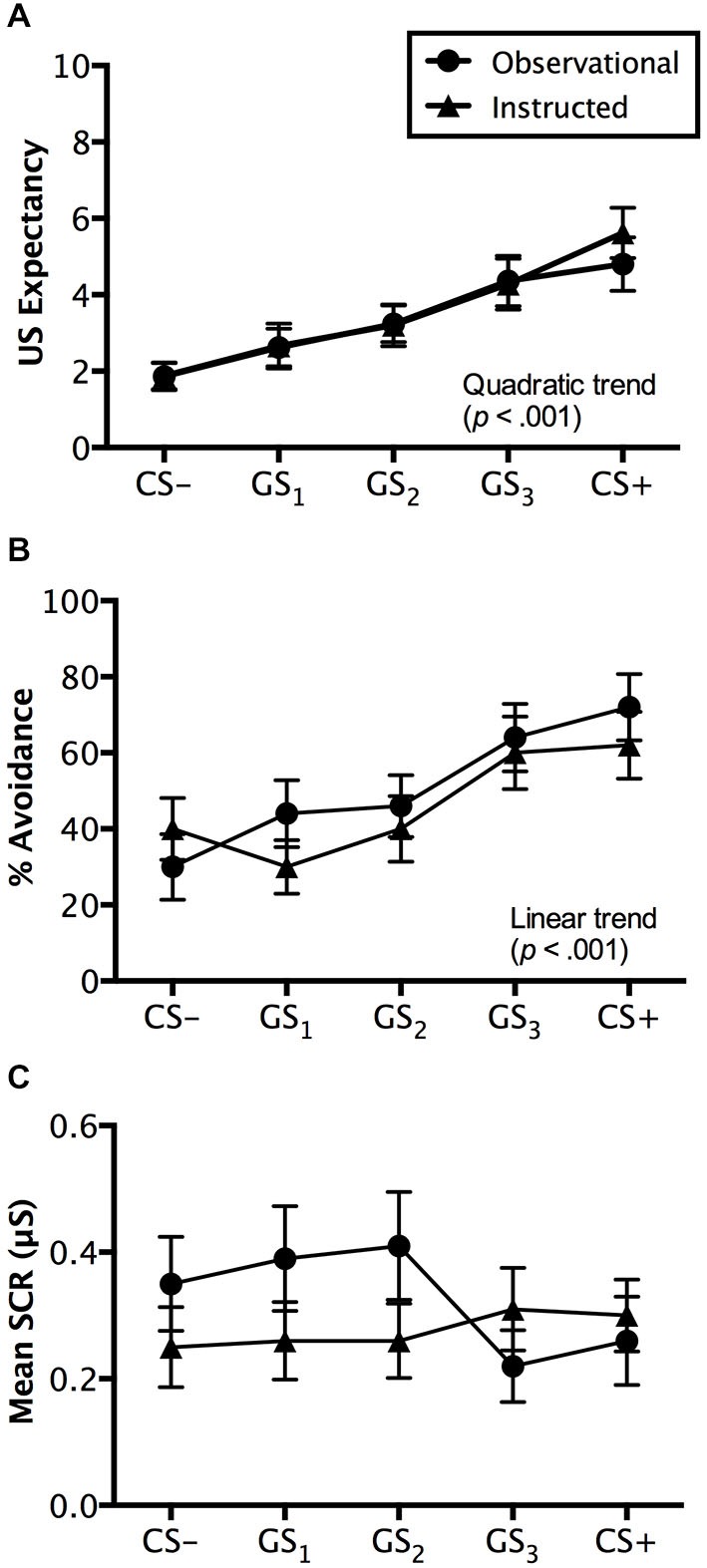
**Generalization test results**. Trial by trial US expectancy **(A)**, proportion of avoidance behavior **(B)**, and mean SCR (μS) (square-root transformed) **(C)** results for conditioning (CS+ and CS−) and generalization stimuli (G_1_, G_2_, G_3_) during generalization testing for the instructed-learning and observational-learning groups (avoidable trials only). Error bars indicate SEM. Linear and/or quadratic terms are also shown.

Ratings made on avoidable trials revealed a significant main effect of stimulus, *F*_(4,192)_ = 18.507, *p* < 0.001, ${\text{η}}_{\text{p}}^{\text{2}}$ = 0.278, BF_01_ = 5.044e-11, with a quadratic increase from CS− to CS+ (*p* < 0.001), but no interaction with group, *F*_(4,192)_ = 0.372, *p* = 0.745, ${\text{η}}_{\text{p}}^{\text{2}}$ = 0.008, BF_01_ = 3.871e-9. Groups did not differ on the ratings they made, *F*_(1,48)_ = 0.071, *p* = 0.791, ${\text{η}}_{\text{p}}^{\text{2}}$ = 0.001, BF_01_ = 4.069, suggesting similar patterns of generalized expectancy (see Figure 4A). Follow-up tests revealed a significant difference between the safety cue, CS−, and the generalized cue it most resembled, GS_1_ (*p* < 0.05). Similar differences in expectancy were found between GS_2_ and GS_3_ (*p* < 0.01) and GS_3_ and CS+ (*p* < 0.05). Mean ratings made to GS_2_ were not significantly greater than those to GS_1_ (*p* = 0.205) (Figure 4A).

Ratings on unavoidable trials displayed a similar pattern with a main effect of stimulus, *F*_(5,240)_ = 39.457, *p* < 0.001, ${\text{η}}_{\text{p}}^{\text{2}}$ = 0.451, BF_01_ = 8.452e-27, as well as both linear (*p* < 0.01) and quadratic trends (*p* < 0.001) found in the generalization gradient, but no interaction with group, *F*_(5,240)_ = 2.173, *p* = 0.084, ${\text{η}}_{\text{p}}^{\text{2}}$ = 0.043, BF_01_ = 1.102e-26. However, a marginally significant difference was found between groups, *F*_(1,48)_ = 3.738, *p* = 0.059, ${\text{η}}_{\text{p}}^{\text{2}}$ = 0.072, BF_01_ = 1.057. Pairwise comparisons revealed that the instructed-learning and observational-learning groups did differ in ratings made during CS+ trials (*p* < 0.01), with higher ratings from the instructed-learning group, but no difference for CS− (*p* = 0.347), GS_1_ (*p* = 0.286), GS_2_ (*p* = 0.800), or GS_3_ trials (*p* = 0.107). The differences between groups on CS+ unavoidable trials likely stems from the different number of directly experienced shock deliveries during avoidance learning. For the observational-learning group, ratings made on CS+ and GS_3_ trials did not differ (*p* = 0.376), but did differ in the instructed-learning group (*p* < 0.05). Similarly, there was no difference in ratings to GS_3_ and GS_2_ in the observational-learning group (*p* = 0.061), but a difference was found in ratings made by the instructed-learning group (*p* < 0.001). Ratings on CS− and GS_1_ trials did not differ for either the instructed-learning (*p* = 0.598) or observational-learning (*p* = 0.792) group, but ratings to GS_1_ and GS_2_ did differ for both groups (instructed-learning: *p* < 0.01; observational-learning: *p* < 0.001).

#### Avoidance Behavior

Avoidance evoked by generalization test stimuli was significantly different, *F*_(4,192)_ = 12.839, *p* < 0.001, ${\text{η}}_{\text{p}}^{\text{2}}$ = 0.211, BF_01_ = 2.560e-7, with a linear trend increase in avoidance from CS− to CS+ (*p* < 0.001), but no interaction with group, *F*_(4,192)_ = 1.230, *p* = 0.301, ${\text{η}}_{\text{p}}^{\text{2}}$ = 0.025, BF_01_ = 4.677e-6. This reflects no differences in avoidance between the instructed-learning and observational-learning groups, *F*_(1,48)_ = 0.248, *p* = 0.620, ${\text{η}}_{\text{p}}^{\text{2}}$ = 0.005, BF_01_ = 3.048. Pairwise comparisons revealed no differences between avoidance evoked by CS− and GS_1_ (*p* = 0.671), G1 and GS_2_ (*p* = 0.263), and GS_3_ and CS+ (*p* = 0.169), but significantly higher levels of avoidance to GS_3_ than GS_2_ (*p* < 0.01). These results suggest a shallow generalization gradient from CS− to GS_2_, but a steep incline from GS_2_ to GS_3_, which then flattened between GS_3_ and CS+ (see Figure 4B).

#### SCR

Results from avoidable trials showed no main effect of stimulus, *F*_(4,160)_ = 1.284, *p* = 0.278, ${\text{η}}_{\text{p}}^{\text{2}}$ = 0.031, BF_01_ = 10.339, no interaction, *F*_(4,160)_ = 1.822, *p* = 0.127, ${\text{η}}_{\text{p}}^{\text{2}}$ = 0.044, BF_01_ = 40.510, and no significant differences between groups, *F*_(1,40)_ = 1.810, *p* = 0.186, ${\text{η}}_{\text{p}}^{\text{2}}$ = 0.043, BF_01_ = 1.777 (see Figure 4C).

Results from unavoidable trials also produced no significant effects of either stimulus type, *F*_(4,160)_ = 0.251, *p* = 0.909, ${\text{η}}_{\text{p}}^{\text{2}}$ = 0.006, BF_01_ = 49.218, group, *F*_(1,40)_ = 1.094, *p* = 0.302, ${\text{η}}_{\text{p}}^{\text{2}}$ = 0.027, BF_01_ = 1.883, or any interaction, *F*_(1,40)_ = 1.240, *p* = 0.296, ${\text{η}}_{\text{p}}^{\text{2}}$ = 0.030 BF_01_ = 647.127.

### Reinstatement Test

#### US Expectancy Ratings

**Figure 5:**
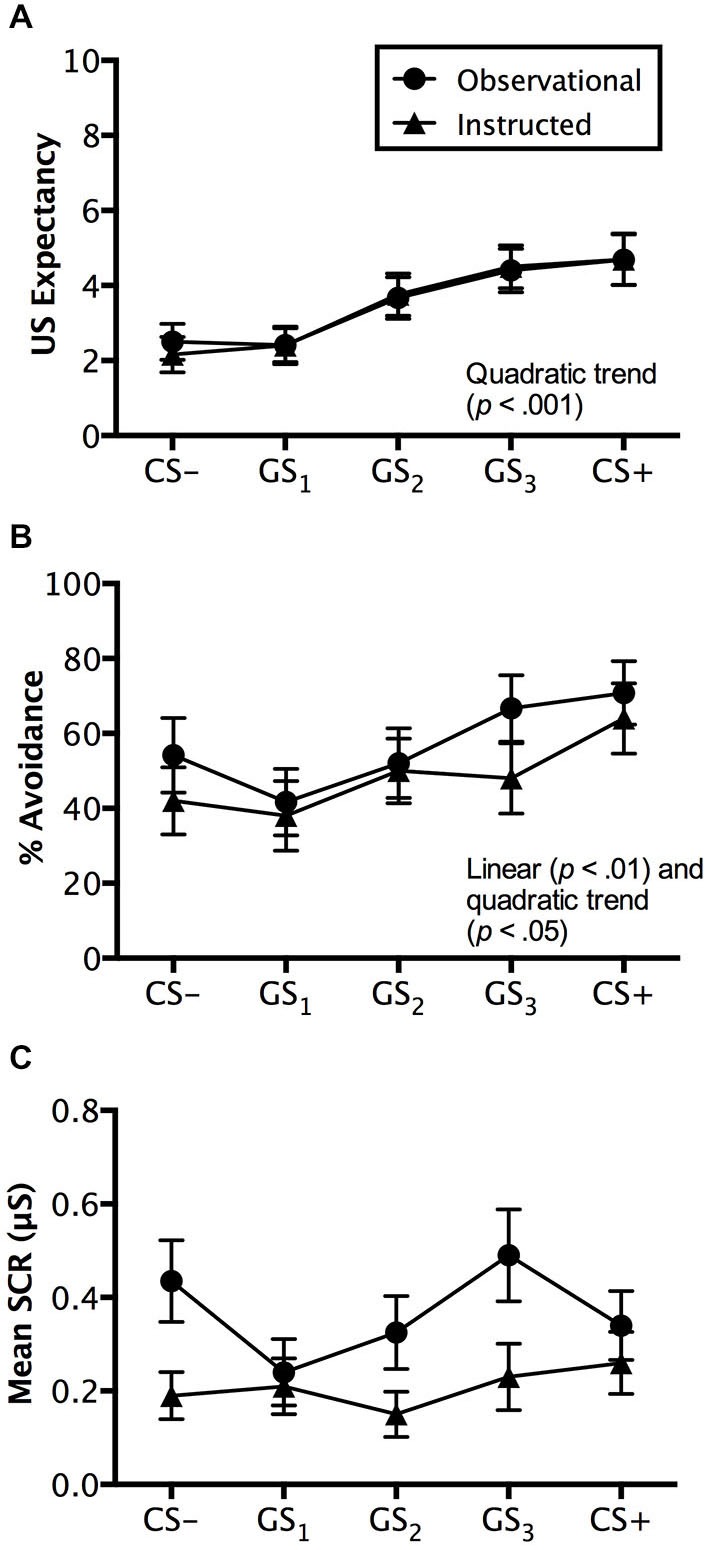
**Reinstatement test results**. Trial by trial US expectancy **(A)**, proportion of avoidance behavior **(B)**, and mean SCR (μS) (square-root transformed) **(C)** results for conditioning (CS+ and CS−) and generalization stimuli (G_1_, G_2_, G_3_) during reinstatement testing for the instructed-learning and observational-learning groups (avoidable trials only). Error bars indicate SEM. Linear and/or quadratic terms are also shown.

Analysis of avoidable trials revealed a significant main effect of stimulus type, *F*_(4,192)_ = 15.110, *p* < 0.001, ${\text{η}}_{\text{p}}^{\text{2}}$ = 0.239, BF_01_ = 5.99e-90, characterized by a quadratic trend (*p* < 0.001), but no interaction with group, *F*_(4,192)_ = 0.139, *p* = 0.9013, ${\text{η}}_{\text{p}}^{\text{2}}$ = 0.003, BF_01_ = 5.704e-7. The instructed-learning and observational-learning groups did not differ in their expectancy ratings during this phase, *F*_(1,48)_ = 0.048, *p* = 0.827, ${\text{η}}_{\text{p}}^{\text{2}}$ = 0.001, BF_01_ = 3.617. Pairwise comparisons revealed that CS+ and GS_3_ (*p* = 0.437) and CS− and GS_1_ (*p* = 0.907) were rated similarly, but significantly higher ratings were seen to GS_2_ over GS_1_ (*p* < 0.001) and GS_3_ over GS_2_ (*p* < 0.05), indicating generalization from both CS+ and CS− to stimuli physically closest on the continuum (see Figure 5A).

Analysis of unavoidable trials displayed a similar pattern; the stimuli presented evoked differential levels of expectancy, *F*_(5,240)_ = 14.324, *p* < 0.001, ${\text{η}}_{\text{p}}^{\text{2}}$ = 0.230, BF_01_ = 2.187e-10, with a quadratic trend (*p* < 0.001), and no interaction with group, *F*_(5,240)_ = 0.438, *p* = 0.757, ${\text{η}}_{\text{p}}^{\text{2}}$ = 0.009, BF_01_ = 1.672e-8. Similarly, the groups did not differ, *F*_(1,48)_ = 0.865, *p* = 0.357, ${\text{η}}_{\text{p}}^{\text{2}}$ = 0.018, BF_01_ = 2.792, and neither ratings of CS+ and GS_3_ (*p* < 0.001) nor CS− and GS_1_ (*p* < 0.05) differed. However, there were significant differences between GS_2_ and GS_3_ (*p* < 0.001) and GS_2_ and GS_1_ (*p* < 0.05), demonstrating similar expectancy ratings to stimuli most physically similar to the CS− and to CS+ respectively, but not for stimuli too dissimilar or far removed from both CS+ and CS− (i.e., GS_2_).

#### Avoidance Behavior

Consistent with the generalization test phase, analysis of avoidance revealed a significant main effect of stimulus, *F*_(4,192)_ = 5.599, *p* < 0.01, ${\text{η}}_{\text{p}}^{\text{2}}$ = 0.104, BF_01_ = 0.013, no interaction, *F*_(4,192)_ = 0.586, *p* = 0.606, ${\text{η}}_{\text{p}}^{\text{2}}$ = 0.012, BF_01_ = 0.529, and no differences between groups, *F*_(1,48)_ = 0.385, *p* = 0.538, ${\text{η}}_{\text{p}}^{\text{2}}$ = 0.008, BF_01_ = 2.653. However, unlike the generalization test phase, both linear (*p* < 0.01) and quadratic (*p* < 0.05) increases in avoidance from CS− to CS+ were found. Pairwise comparisons revealed no significant difference between CS− and GS_1_ (*p* = 0.104) or between GS_2_ and GS_3_, (*p* = 0.304), but a significant difference between GS_1_ and GS_2_ (*p* < 0.05) and GS_3_ to CS+ (*p < 0*.05), which indicates a shift in avoidance from GS_2_ towards the CS+, while the GS_3_ generalization gradient became steeper towards CS+ (see Figure 5B).

#### SCR

Analysis of avoidable trials revealed no main effect of stimulus type, *F*_(4,152)_ = 1.961, *p* = 0.103, ${\text{η}}_{\text{p}}^{\text{2}}$ = 0.049, BF_01_ = 4.168, and no interaction with group, *F*_(4,152)_ = 1.661, *p* = 0.162, ${\text{η}}_{\text{p}}^{\text{2}}$ = 0.042, BF_01_ = 5.398. Interestingly, a significant difference between groups was found, *F*_(1,38)_ = 5.219, *p* < 0.05, ${\text{η}}_{\text{p}}^{\text{2}}$ = 0.121, BF_01_ = 0.435, which pairwise comparisons suggested was driven by differences in SCR amplitude to CS− GS_2_, and GS_3_ (all *p*’s < 0.05), but not to GS_1_ (*p* = 0.795) or CS+ (*p* = 0.406). Overall, it appeared that the observational-learning group produced consistently higher SCRs to all stimuli (Figure 5C).

For the unavoidable trials, analysis revealed a significant main effect of stimulus type, *F*_(4,152)_ = 3.485, *p* < 0.01, ${\text{η}}_{\text{p}}^{\text{2}}$ = 0.084, BF_01_ = 0.536, an interaction with group, *F*_(4,152)_ = 3.148, *p* < 0.05, ${\text{η}}_{\text{p}}^{\text{2}}$ = 0.077, BF_01_ =.031, and significant differences between the groups, *F*_(1,38)_ = 8.258, *p* < 0.01, ${\text{η}}_{\text{p}}^{\text{2}}$ = 0.179, BF_01_ = 0.169. SCRs differed between groups to CS−, GS_2_, and CS+ (all *p*’s < 0.05), with higher SCRs elicited by the observational-learning than the instructed-learning group, but not to GS_1_ (*p* = 0.222) or GS_3_ (*p* = 0.923).

### Return of Fear: Comparing Generalization and Reinstatement Tests

To assess return of fear, the final presentation of each stimulus in the generalization test phase was compared to the first presentation following US reinstatement. Repeated measures ANOVA was run with group as the between groups variable and trial order as the within subjects factor. Table 3 shows the mean US expectancy ratings, proportion of avoidance, and SCR for all stimuli presented during the generalization and reinstatement test phases.

**Table 3:** **Means (and standard deviation) US expectancy ratings, proportion of avoidance and SCR for CS-, GS_1_, GS_2_, GS_3_ and CS+ during avoidable trials in the generalization test and reinstatement test phases for the instructed-learning (instructed) and observational-learning (observed) groups**.

Stimulus	Group	Generalization test			Reinstatement test		
		Ratings	SCR	% Avoidance	Ratings	SCR	% Avoidance
CS−	Instructed	1.88 (1.77)	0.49 (0.58)	40 (40.82)	2.16 (2.36)	0.39 (0.44)	42 (44.91)
	Observed	1.86 (1.74)	0.71 (0.67)	30 (43.30)	2.40 (2.36)	0.87 (0.76)	52 (48.90)
GS_1_	Instructed	2.66 (2.46)	0.53 (0.56)	30 (35.36)	2.40 (2.50)	0.43 (0.52)	38 (46.28)
	Observed	2.62 (2.93)	0.77 (0.76)	44 (44.06)	2.22 (2.21)	0.48 (0.63)	40 (43.30)
GS_2_	Instructed	3.20 (2.71)	0.53 (0.53)	40 (43.30)	3.76 (2.81)	0.29 (0.43)	50 (43.30)
	Observed	3.24 (2.39)	0.81 (0.77)	46 (40.62)	3.76 (2.80)	0.66 (0.69)	50 (45.64)
GS_3_	Instructed	4.28 (3.34)	0.61 (0.60)	60 (47.87)	4.50 (2.85)	0.46 (0.61)	48 (46.73)
	Observed	4.36 (3.29)	0.43 (0.53)	64 (44.53)	4.22 (2.96)	0.98 (0.87)	64 (44.53)
CS+	Instructed	5.62 (3.28)	0.59 (0.52)	62 (43.97)	4.70 (3.44)	0.51 (0.58)	64 (46.82)
	Observed	4.80 (3.50)	0.93 (0.63)	72 (43.49)	4.50 (3.35)	0.67 (0.65)	68 (3.01)

#### US Expectancy Ratings

Results revealed a significant main effect of trial order, *F*_(9,423)_ = 12.953, *p* < 0.001, ${\text{η}}_{\text{p}}^{\text{2}}$ = 0.216, BF_01_ = 3.283e-16, but no interaction between group and trial, *F*_(9,423)_ = 0.568, *p* = 0.744, ${\text{η}}_{\text{p}}^{\text{2}}$ = 0.012, BF_01_ = 1.091e-13, and no differences between groups, *F*_(1,47)_ = 0.149, *p* = 0.702, ${\text{η}}_{\text{p}}^{\text{2}}$ = 0.003, BF_01_ = 4.031. Ratings did not differ between groups for the CS− (*p* = 0.143), GS_1_ (*p* = 0.579), or CS+ (*p* = 0.963), but there was a significant increase in ratings to GS_2_ (*p* < 0.01), and a near significant decrease for GS_3_ (*p* = 0.051) from generalization test to reinstatement test. These results indicate a return of fear towards more ambiguous stimuli, but stable responding to those with a prior history of either shock or no shock, which generalized only to the most physically similar stimuli (i.e., GS_1_ and GS_3)_.

Results from the unavoidable trials followed a similar pattern: a significant main effect of trial, *F*_(11,495)_ = 17.360, *p* < 0.001, ${\text{η}}_{\text{p}}^{\text{2}}$ = 0.278, BF_01_ = 3.149e-27, no interaction between group and trial, *F*_(11,495)_ = 0.553, *p* = 0.798, ${\text{η}}_{\text{p}}^{\text{2}}$ = 0.012, BF_01_ = 5.732e-25, and no difference between groups, *F*_(1,45)_ = 2.920, *p* = 0.094, ${\text{η}}_{\text{p}}^{\text{2}}$ = 0.061, BF_01_ = 1.397. These results indicate that in the absence of the availability of the avoidance response, no change in US expectancy ratings in either the instructed-learning or observational-learning groups occurred following US reinstatement.

#### Avoidance Behavior

When testing for return of avoidance, we found a significant main effect of trial order, *F*_(9,423)_ = 4.720, *p* < 0.001, ${\text{η}}_{\text{p}}^{\text{2}}$ = 0.091, BF_01_ = 4.386e-4, no interaction, *F*_(9,423)_ = 0.568, *p* = 0.644, ${\text{η}}_{\text{p}}^{\text{2}}$ = 0.015, BF_01_ = 0.077, and no difference between groups, *F*_(1,47)_ = 0.388, *p* = 0.537, ${\text{η}}_{\text{p}}^{\text{2}}$ = 0.008, BF_01_ = 3.288. There was a significant increase in avoidance responding to CS− (*p* < 0.05), but no change to GS_1_ (*p* = 0.375), GS_2_ (*p* = 0.963), GS_3_ (*p* = 0.129), or CS+ (*p* = 0.980).

#### SCR

The analysis of SCR during avoidable trials revealed no main effect, *F*_(9,342)_ = 1.462, *p* = 0.161, ${\text{η}}_{\text{p}}^{\text{2}}$ = 0.037, BF_01_ = 20.990, no interaction, *F*_(9,342)_ = 0.938, *p* = 0.492, ${\text{η}}_{\text{p}}^{\text{2}}$ = 0.024, BF_01_ = 1128.745, and no significant differences between groups, *F*_(1,38)_ = 1.894, *p* = 0.177, ${\text{η}}_{\text{p}}^{\text{2}}$ = 0.047, BF_01_ = 2.375. There was a significant increase in SCR during presentations of CS− (*p* < 0.05) and GS_3_ (*p* < 0.05), but no significant change to presentations of GS_1_ (*p* = 0.805), GS_2_ (*p* = 0.912) and CS+ (*p* = 0.666).

Analysis of unavoidable trials revealed no main effect, *F*_(9,342)_ = 1.481, *p* = 0.183, ${\text{η}}_{\text{p}}^{\text{2}}$ = 0.038, BF_01_ = 19.863, no interaction, *F*_(9,342)_ = 1.165, *p* = 0.325, ${\text{η}}_{\text{p}}^{\text{2}}$ = 0.030, BF_01_ = 384.817, and no difference between groups *F*_(1,38)_ = 3.581 *p* = 0.066, ${\text{η}}_{\text{p}}^{\text{2}}$ = 0.086, BF_01_ = 1.551, indicating no change in physiological responding for both groups following US reinstatement in the absence of avoidance.

### Shapes of Generalization Gradients

To assess the shape of the generalization gradient, we adopted the method of *linear departure* described by van Meurs et al. (2014) to determine the extent to which the gradients departed from linearity: *(average GS_1_, GS_2_, GS_3_)−(average CS+, CS−)*. The average of the CS+ and CS− reflects the directly trained midpoint of the generalization gradient (*overall avoidance*) and the average responses of the GSs (*maladaptive avoidance*) could fall either above (positive departure) or below (negative departure) this midpoint. For the combined sample only (i.e., both groups), a significant positive correlation was found between gradients of avoidance behavior and US expectancy ratings (*r* = 0.515, *p* < 0.001), but not SCR (*r* = 0.168, *p* = 0.287) during the reinstatement test phase only. No significant correlations between avoidance and either SCR (*p* = 0.670) or US expectancy ratings (*p* = 0.389) was found during the generalization test phase.

## Discussion

The aim of the present study was to compare, for the first time, instructed-learning and observational-learning pathways of avoidance on generalized avoidance behavior in humans. Participants first underwent fear conditioning and were then divided into two groups that differed in how avoidance was acquired (either through instructions about the avoidance response for the instructed-learning group or through watching a video recording of a demonstrator performing the avoidance response for the observational-learning group). Both groups were then tested in extinction for generalization of avoidance to stimuli physically resembling the CS+ along the formal dimension of size. Return of fear and avoidance was then tested in a reinstatement phase after unsignaled US presentations. Results showed that groups did not differ by the end of fear conditioning, with each group showing enhanced expectancy of shock following CS+ relative to CS−, although SCR measures fell short of statistical significance for this phase. Results also showed that both groups demonstrated comparable levels of US expectancy, avoidance behavior and physiological measures in a gradient-like manner from CS+ across the generalization stimuli to CS−. Return of fear was evident during reinstatement testing, suggesting that generalized avoidance remains persistent following the completion of extinction testing. Taken together, these findings are the first demonstration of similar generalization gradients of a signaled operant avoidance response following instructed and observational learning. We will now discuss these findings and the limitations of the present study in more detail below.

### Instructed-Learning and Social Transmission of Generalized Avoidance

Previous studies have shown that fear learning may be acquired vicariously via instructions and social observation in both adults (Olsson and Phelps, 2004, 2007; Mechias et al., 2010) and children (e.g., Askew and Field, 2007; Reynolds et al., 2014). Effects of vicarious learning on fear related cognitions and approach-avoidance behavior has also been reported with children (Broeren et al., 2011). The present study is the first however to directly contrast Rachman’s (1977) social observation pathway with instructed avoidance with adults, and to examine generalization of avoidance established via these pathways. Participants in the instructed-learning group were informed about the availability of the avoidance response, which was cued by an onscreen border, the avoidance response was fully described, and the CS+ and CS− were presented in random order a fixed number of times. These procedures therefore likely employed a combination of instructed and instrumental learning processes (Raes et al., 2014). Some studies have shown that explicit instructions about the avoidance response are not necessary for successful acquisition of avoidance, even in studies requiring multiple acquisition sessions to maintain a predetermined training criterion (e.g., Sheynin et al., 2014). In the present study, some form of instruction about avoidance was deemed necessary to facilitate comparison with avoidance acquired via social learning. It is likely therefore that acquisition of avoidance in the instructed-learning group could potentially have been a mismatch with the contingencies experienced by the observational-learning group who passively viewed a movie of a demonstrator performing the correct avoidance response. Future research would be well advised to determine whether or not the task instructions given to the instructed-learning group were either necessary or sufficient for the acquisition of avoidance and, if so, what effects it may have had on subsequent generalization. Moreover, a demonstration of generalized avoidance following trial and error instrumental learning with minimal or no instructions about the avoidance response would also be salutary (Dymond et al., 2012).

Both groups were exposed to an identical generalization test where the effects of the different avoidance pathways were tested. Findings indicated that both groups responded in a similar manner during the generalization test phase, with avoidance behavior and US expectancy ratings falling along a generalization gradient of responding (van Meurs et al., 2014). In both groups, the generalization cue G_3_ elicited similar expectancy ratings and levels of avoidance to CS+, with less pronounced differences seen in SCR (Figure 4). This cue was physically closest to CS+ along the continuum of size but had never been paired with shock. Yet, it elicited levels of fear and prompted actual avoidance behavior in a manner resembling a directly learned danger cue. Although G_3_ and the other generalization stimuli lacked a direct conditioning history with shock, their presentation during the generalization test was sufficient to prompt maladaptive avoidance behavior by both groups as participants clearly adopted a “better safe than sorry” approach (Lommen et al., 2010). That is, rather than wait to see whether or not withholding avoidance in the presence of the GSs would be followed by shock, a significant proportion of avoidance behavior was seen and a high expectancy of shock was simultaneously recorded. Taken together, these findings suggest that participants in both groups had a high expectancy that shock would follow nonavoidance, which motivated the levels of avoidance behavior seen. This is the first such demonstration of generalized avoidance in a signaled operant task without additional, ongoing task conflict (e.g., approach-avoidance conflict; van Meurs et al., 2014).

The present findings support the use of analyses of the slope of individual participant’s generalization gradients to determine the extent to which they depart from linearity. We adopted the van Meurs et al. (2014) method of calculating *linear departure* to describe the shape of the gradient where the mean of the CS+ and CS− reflects the directly trained midpoint and where the mean of the GSs could fall either above (positive departure) or below (negative departure) this midpoint. Similar to van Meurs et al. (2014) we found correlations between our measures of generalized avoidance, with the slope of participants’ expectancy ratings positively correlating with the proportion of avoidance in the reinstatement test phase only. The absence of these correlations in the generalization test phase may indicate either early effects of extinction or, as will be discussed below, inadequate power in the number of stimulus presentations of the GS’s used to calculate departure.

### Reinstatement of Generalized Fear and Avoidance

As described above, reinstatement or return of fear (and avoidance) was tested by unsignaled US presentations following the extinction generalization test, which was then repeated. Reinstatement research with humans is still very much in its infancy (Haaker et al., 2014) and the present study represents the first such investigation in the context of avoidance generalization. As might be expected, a brief reintroduction of fear induced avoidance behavior, expectancy of shock and SCR but not at levels significantly different from earlier (Figure 5). In fact, our analyses indicated that reinstatement testing boosted levels of maladaptive responding but only to the CS− across all performance measures, suggesting it was deemed a potential threat. Such a finding has been observed previously in the context of fear learning and extinction (Kull et al., 2012), but this is the first such demonstration in a study of avoidance generalization in healthy humans. It remains to be seen whether the reported transient effects of reinstatement and its susceptibility to methodological factors such as a stimulus sequence effects (Haaker et al., 2014) are observed in other avoidance learning pathways and generalization paradigms (see also Mertens et al., 2015).

### Social Observational Pathway to Generalized Avoidance

It is well known that nonhumans can acquire fears via observation. For instance, Mineka et al. (1984) had observer monkeys without snake-fear watch their model parent monkeys interact with real, toy and model snakes. Five of the six observer monkeys readily acquired fear and avoidance of snakes, which generalized to snake-related stimuli and novel contexts. It is also relatively well known that human adults and infants can acquire fear vicariously via social observation. For example, in a neuroimaging study, Olsson et al. (2007) showed participants a movie of another person experiencing fear and distress when receiving shocks paired with a CS+. These authors found that similar neural systems were recruited during acquisition (observation) and expression (test) of learned fear, highlighting a common neurobehavioral mechanism supporting directly learned and observed fear pathways. Moreover, facial fear expression readily functions as a US in human adults (Vaughan and Lanzetta, 1980) and nonhumans (Mineka et al., 1984). Behavioral research has also highlighted similar findings in typically developing young infants. Broeren et al. (2011), for example, exposed young children to a peer modeling intervention in which they viewed either positive or negative modeling films showing peers approach the same wooden box used in their behavioral approach/avoidance task. They found that positive modeling decreased avoidance tendencies towards known and unknown animals, while negative modeling had little effect on avoidance of the modeled animal but did decrease avoidance tendencies towards the non-modeled animal.

The present findings are the first to show that avoidance may initially be acquired via observational-learning and then subsequently generalize to exemplars perceptually related to the conditioned danger cue in a manner resembling that seen in the generalization of instructed-learning of avoidance. This is, therefore, the first study to investigate avoidance behavior as both the acquisition pathway of comparison and the means of testing potential similarities between pathways during a common generalization test. Previous studies have tended to compare different pathways to fear or to use avoidance as a one-off behavioral outcome of fear; the present study is unique then for its emphasis on both avoidance acquisition and generalization.

The present paradigm affords several opportunities to further investigate generalized avoidance and the social transmission of avoidance. First, our paradigm may prove useful in detecting social learning effects on acquisition and generalization of avoidance. For instance, varying the expressive details, accuracy or racial group membership of the facial expression modeled (e.g., Golkar et al., 2015) may influence the persistence of avoidance and might even produce pronounced effects with fear relevant stimuli in individuals with and without an anxiety disorder. Second, extending the observational phase to include modeling of unsignaled US presentations prior to a reinstatement test phase would be a novel synthesis of observational-learning of avoidance with human reinstatement research and allow for the detection of potentially transient effects on generalization. Third, exposing participants to a movie where the US is removed (extinction) or where the avoidance response is prevented and the US presented independent of responding (Higgins and Morris, 1984) would permit an examination of the relative effectiveness of these separate, operant extinction methods. Moreover, effects of presenting either the generalized or learned cues in tandem with these extinction methods could be tested and applied to analog analyses of exposure-based therapy for reducing levels of problematic avoidance that is often seen in anxiety disorders. Fourth, once further validated, the present paradigm may prove useful in identifying the neurobehavioral mechanisms of avoidance generalization and testing for potential differences in generalization in those with and without an anxiety disorder.

### Limitations

A limitation of the present study was the failure to detect significant effects of stimulus type in SCR during fear conditioning and subsequent generalization test phases. The Bayesian analysis conducted of SCR data obtained during fear conditioning indicated that the data were insensitive in distinguishing the alternative hypothesis from the null. This may be related to the small sample size, the loss of some SCR data, and the number of trials presented in the generalization test and reinstatement test phases. This was exacerbated by the use of both avoidable and unavoidable trials for CSs and GSs, which meant the number of analyzed trials was reduced by more than 50%. The design of the present task also meant that trials involving ΔCS− were never avoidable, and consequently no data for these trials were included in our final analysis. The inclusion of avoidable and unavoidable trials was intended as a form of within-subject contrast to help ensure reliable acquisition of discriminated avoidance (for the instructed-learning group and which was observed indirectly for the observational-learning group) and to maintain generalized avoidance when the US was withheld in extinction and reinstatement testing. A limitation of this approach was that our design did not allow for analysis of both avoidable and unavoidable trials of the ΔCS−, which we might predict would evoke a low level of generalized avoidance comparable to the CS−. Indeed, the reported difference in CS+ ratings on unavoidable trials may have resulted from the fact that the instructed group received four more unavoidable shocks than the observational group during avoidance learning. Further research on the relative roles of avoidable and unavoidable trials on the generalization of avoidance is therefore warranted.

The generalization test phase was also an extinction test since the US was withheld on all trials. Previous work on fear generalization (see Dymond et al., in press) and avoidance generalization (van Meurs et al., 2014) has tended to employ variants of the “steady state” generalization test by continuing to present the US on some trials because doing so prevents extinction and gives participants the opportunity to learn that the CS+ is still dangerous and the GSs are, at least putatively, safe. Because we were interested in reinstatement of avoidance, we chose to conduct generalization testing in extinction; future research should investigate other paradigms to probe for generalization that continue to present the US on some trials.

An additional limitation is that performing the avoidance response may have influenced SCR recording during the test phases. Since the SCR interval analyzed was 6 s post-stimulus onset, the peak SCR may not have occurred until after the avoidance response was made. It is possible therefore that performing the avoidance response within the 6 s interval reduced peak amplitude SCR, but would not have influenced US expectancy ratings, which were made before avoidance responding. This was reflected during the reinstatement test phase during unavoidable trials, where US expectancies and SCR responding to CS+ remained higher than CS−. In the absence of avoidance we observed differences in SCR to CS’s and GS’s, which diminished in the presence of avoidance. As a result, the current study does not allow strong claims to be made regarding the physiological responding of avoidance generalization. In future research, the avoidance response should ideally be separated from SCR recording until such a time that the peak SCR is recorded and additional physiological measures, such as fear-potentiated startle, incorporated into the analysis of generalized avoidance.

Finally, the results of the present study would be strengthened by undertaking tests for retrospective identification of the CS+ and determining the extent to which participants discriminated between the danger cue and the cue it most resembled (i.e., GS_3_). Also, employing a greater number of generalization stimuli, which could be combined to make classes of GSs (Lissek et al., 2008), may serve to facilitate potentially larger perceptual generalization differences between the cues. Accurate and high post-experimental recognition of the CS+ would thereby confirm both discrimination of danger and safety and ensure that the generalization gradient obtained was the one intended by the experimenter.

## Conclusions

The present study demonstrated, for the first time, the equivalence of instructed-learning and observational-learning pathways of avoidance acquisition on the generalization of avoidance behavior. After fear conditioning, groups either were instructed that a simple instrumental response in the presence of the CS+ cancelled upcoming shock or observed a short movie showing a demonstrator in the same experimental context performing the avoidance response to prevent shock. In two test phases, in the absence of the US, danger and safety cues were first presented along with GSs and a profile consisting of avoidance behavior, US expectancy and SCR measured. Return of fear was then probed in a reinstatement test following unsignaled US presentations. Findings revealed a generalization gradient in responding with the greatest proportion of avoidance and fear expectancy elicited by the CS+, with decreasing levels of avoidance, fear and SCR to the GSs of decreasing similarity to the CS+. Reinstatement testing demonstrated that generalized avoidance remained remarkably intact following a brief reintroduction of fear. The present findings show that generalized avoidance is a resilient behavioral consequence of fear learning and may emerge in the absence of a direct avoidance learning history. These findings also contribute to the literature on alternatives to frequentist statistical inference approaches by reporting a Bayesian analysis as an alternative to null hypothesis significance testing (NHST; Wagenmakers, 2007; Masson, 2011; Jarosz and Wiley, 2014). A non-significant result cannot provide evidence against the alternative hypothesis but is regularly used in such a way (Dienes, 2014). Similar to previous analyses (Krypotos et al., 2011, 2014), we used Bayesian analysis to determine if the absence of any differences between our groups supported the null hypothesis over the alternative hypothesis, which is not possible using conventional NHST.

## Conflict of Interest Statement

The authors declare that the research was conducted in the absence of any commercial or financial relationships that could be construed as a potential conflict of interest.
